# The impact of non-alcoholic fatty liver disease and metabolic syndrome on the progression of coronary artery calcification

**DOI:** 10.1038/s41598-018-30465-y

**Published:** 2018-08-13

**Authors:** Yun Kyung Cho, Yu Mi Kang, Jee Hee Yoo, Jiwoo Lee, Seung Eun Lee, Dong Hyun Yang, Joon-Won Kang, Joong-Yeol Park, Chang Hee Jung, Hong-Kyu Kim, Woo Je Lee

**Affiliations:** 1 Department of Internal Medicine, Asan Medical Center, University of Ulsan College of Medicine, Seoul, Republic of Korea; 2 International Healthcare Center, Asan Medical Center, University of Ulsan College of Medicine, Seoul, Republic of Korea; 3 Department of Internal Medicine, Dongguk University Ilsan Hospital, Dongguk University College of Medicine, Goyang, Republic of Korea; 4 Department of Radiology, Asan Medical Center, University of Ulsan College of Medicine, Seoul, Republic of Korea; 5 Department of Health Screening and Promotion Center, Asan Medical Center, University of Ulsan College of Medicine, Seoul, Republic of Korea

## Abstract

It is unclear whether non-alcoholic fatty liver disease (NAFLD) is an independent risk factor for cardiovascular disease. We examined the independent impact of NAFLD on the progression of the coronary artery calcification (CAC) score, a well-known marker of atherosclerosis progression. We examined 1,173 asymptomatic participants who underwent repeated CAC score measurement during routine health examinations. The subjects were categorised into four groups based on the presence (+) or absence (−) of NAFLD and metabolic syndrome (MetS). The progression of CAC score was defined as either incident CAC in a CAC-free population at baseline or an increase of ≥2.5 units between the baseline and the final square roots of the CAC scores of participants with detectable CAC at baseline. CAC progression was seen in 18.6% (98/526), 28.3% (77/272), 29.1% (30/103) and 32.0% (87/272) of the subjects with NAFLD(−)/MetS(−), NAFLD(+)/MetS(−), NAFLD(−)/MetS(+) and NAFLD(+)/MetS(+), respectively. The subjects with NAFLD(+)/MetS(+) and NAFLD(+)/MetS(−) had a significantly higher risk of CAC progression than those with NAFLD(−)/MetS(−) (multivariate-adjusted odds ratio [OR]: 1.76; 95% confidence interval [CI]: 1.18–2.62 and multivariate-adjusted OR: 1.53, 95% CI: 1.05–2.23, respectively). NAFLD is an independent risk factor for CAC progression, irrespective of the presence of MetS.

## Introduction

Non-alcoholic fatty liver disease (NAFLD) is emerging as the leading cause of chronic liver disease worldwide^1^. Furthermore, NAFLD is not only associated with liver-related morbidity and mortality, but also with serious extrahepatic complications, including cardiovascular disease (CVD)^2^. However, the importance of NAFLD as an independent contributor to CVD is still uncertain because numerous cardiovascular risk factors are shared by both NAFLD and CVD^2^. In addition, NAFLD has been recognised as the hepatic manifestation of metabolic syndrome (MetS) and insulin resistance^3^. Therefore, whether NAFLD is merely a part of the systemic derangement composing MetS or whether NAFLD is an important CVD risk factor still remains unclear.

The coronary artery calcification (CAC) score, measured by multi-detector computed tomography (MDCT), reflects the overall coronary plaque burden and a high CAC score is independently and incrementally predictive of future coronary events and prognosis^4^. Moreover, recent studies show that CAC score progression is significantly related to higher risk of future CVD events and all-cause mortality^5,6^. Because atherosclerosis is a dynamic process, CAC score progression provides a better reflection of atherosclerosis progression than the baseline CAC score, making the assessment of the effectiveness of medical treatment and the risk of future CVD events possible^5,7^.

A recent meta-analysis demonstrated a significant positive association between NAFLD and CAC, supporting the role of NAFLD as an independent predictor of CVD^8^. However, to date, few studies have evaluated whether NAFLD is longitudinally associated with CAC progression, independently of MetS. In this study, we aimed to investigate the impact of NAFLD and MetS on CAC progression in an asymptomatic, middle-aged, Korean population. To clarify the independent impact of NAFLD on CAC progression, we analysed the risk of CAC progression in subjects divided into four groups based on the presence (+)/absence (−) of NAFLD and MetS.

## Results

### Baseline characteristics of the participants based on the presence of NAFLD and/or MetS

We divided the 1,173 participants (mean age, 54.1 ± 7.4 years; range, 33–79 years) into four sub-groups based on the presence or absence of NAFLD and/or MetS as follows: (1) subjects without either abnormality (n = 526; 44.8%); (2) subjects with NAFLD only (n = 272; 23.2%); (3) subjects with MetS only (n = 103; 8.8%) and (4) subjects with both abnormalities (n = 272; 23.2%). The baseline characteristics of the subjects in each sub-group are shown in Table 1. Overall, males predominated (81.5%). The subjects with NAFLD only or with both abnormalities were more likely to be male. Comparison of these parameters among the groups showed that the NAFLD only group, the MetS only group and the group with both abnormalities had unfavourable metabolic profiles. All three groups had higher body mass index (BMI), waist circumference (WC), triglyceride (TG), fasting plasma glucose (FPG), glycosylated haemoglobin (HbA1c), uric acid, high-sensitive C-reactive protein (hsCRP) and lower high-density lipoprotein cholesterol (HDL-C) than the group without either abnormality. The 10-year Framingham risk score (FRS) and the 10-year atherosclerotic CVD (ASCVD) risk scores in the NAFLD only group were also significantly higher than those in normal subjects (Table 1). The proportion of subjects with hypertension in the MetS only group and the group with both abnormalities was significantly higher than that in the NAFLD only group, although the NAFLD only group demonstrated a higher prevalence of hypertension than the normal subjects (Table 1). The alanine aminotransferase (ALT) level in the NAFLD only group was higher than that in the MetS only group; however, the opposite was true for the gamma-glutamyltransferase (GGT) level (Table 1). The mean age, total cholesterol and low-density lipoprotein cholesterol (LDL-C) and follow-up intervals did not differ among the four groups (Table 1).

**Table 1:** Baseline clinical and biochemical characteristics based on the presence of NAFLD and/or MetS.

	Total	NAFLD(−)MetS(−)	NAFLD(+)MetS(−)	NAFLD(−)MetS(+)	NAFLD(+)MetS(+)	*P*
N (%)	1,173	526 (44.8)	272 (23.2)	103 (8.8)	272 (23.2)	
Age (years)	54.1 ± 7.4	54.1 ± 7.4	53.4 ± 7.1	55.5 ± 8.4	54.4 ± 7.4	0.079
Sex (% male)	81.5	72.2^a^	91.2^b^	79.6^a^	90.4^b^	<0.001
BMI (kg/m^2^)	25.0 ± 3.0	23.5 ± 2.4	25.2 ± 2.4^a^	26.1 ± 4.2^a^	27.1 ± 2.5	<0.001
WC (cm)	87.0 ± 8.2	82.4 ± 7.1	88.2 ± 6.4^a^	90.5 ± 8.1^a^	93.1 ± 6.6	<0.001
Systolic BP (mmHg)	119.5 ± 12.9	116.3 ± 12.4	119.0 ± 11.8	123.6 ± 13.8^a^	124.6 ± 12.5^a^	<0.001
Diastolic BP (mmHg)	76.6 ± 10.6	74.2 ± 10.3	76.5 ± 9.6^a^	78.4 ± 11.4^ab^	80.7 ± 10.5^b^	<0.001
Current smoker (%)	27.4	22.1	29.0^a^	32.0^a^	34.2^a^	<0.001
Moderate drinker (%)	53.1	47.3^a^	54.0^ab^	58.3^b^	61.4^b^	<0.001
Physically active (%)	43.6	48.5^a^	41.9^ab^	47.6^a^	34.6^b^	0.001
Family history of diabetes (%)	24.0	22.2	24.3	20.4	28.7	0.080
Diabetes (n, %)	155 (13.2)	29 (5.5)	40 (14.7)^a^	20 (19.4)^ab^	66 (24.3)^b^	<0.001
Hypertension (n, %)	393 (33.5)	107 (20.3)^a^	65 (23.9)^a^	63 (61.2)^b^	158 (58.1)^b^	<0.001
FPG (mmol/L)	5.8 ± 1.0	5.5 ± 0.8	5.8 ± 1.0^a^	6.0 ± 0.9^ab^	6.3 ± 1.2^b^	<0.001
HbA1c (%)	5.5 (5.3–5.9)	5.4 (5.2–5.7)	5.5 (5.3–5.9)^a^	5.7 (5.4–6.0)^ab^	5.7 (5.5–6.2)^b^	<0.001
Total cholesterol (mmol/L)	5.2 ± 0.8	5.2 ± 0.8	5.2 ± 0.8	5.0 ± 0.8	5.2 ± 0.9	0.070
TG (mmol/L)	1.3 (1.0–1.8)	1.0 (0.8–1.4)	1.4 (1.1–1.7)	1.8 (1.1–2.3)	2.0 (1.4–2.6)	<0.001
LDL-C (mmol/L)	3.3 ± 0.7	3.2 ± 0.7	3.3 ± 0.7	3.1 ± 0.8	3.3 ± 0.8	0.035
HDL-C (mmol/L)	1.3 ± 0.3	1.5 ± 0.3	1.3 ± 0.3^a^	1.2 ± 0.3^ab^	1.1 ± 0.2^b^	<0.001
Uric acid (µmol/L)	345.9 ± 83.0	319.6 ± 76.5	363.2 ± 77.5^ab^	347.4 ± 90.9^a^	378.7 ± 81.5^b^	<0.001
AST (U/L)	25 (22–31)	24 (21–29)^a^	27 (22–34)^bc^	25 (21–30)^ab^	28 (23–35)^c^	<0.001
ALT (U/L)	23 (17–31)	19 (15–24)	27 (19–37)	23 (17–28)	30 (22–41)	<0.001
GGT (U/L)	25 (17–40)	19 (13–30)	28 (19–42)^a^	32 (18–48)^a^	34 (24–49)	<0.001
HsCRP (mg/L)	0.6 (0.3–1.3)	0.5 (0.3–1.1)	0.6 (0.4–1.3)^a^	0.7 (0.4–1.5)^ab^	0.9 (0.5–1.6)^b^	<0.001
10-year FRS (%)	6.0 (3.0–10.0)	5.0 (2.0–8.0)	6.0 (4.0–10.0)^a^	10.0 (4.0–12.0)^ab^	10.0 (6.0–12.0)^b^	<0.001
10-year ASCVD risk score (%)	5.5 (2.7–9.7)	4.0 (1.8–7.5)	5.6 (2.9–9.6)	6.9 (3.6–13.5)^a^	8.5 (5.1–13.1)^a^	<0.001
Baseline CAC score	0.0 (0.0–21.3)	0.0 (0.0–10.0)	0.0 (0.0–19.0)	1.0 (0.0–94.0)^a^	1.0 (0.0–57.5)^a^	<0.001
Last follow-up CAC score	0.6 (0.0–47.9)	0.0 (0.0–26.1)	1.5 (0.0–47.2)	7.0 (0.0–146.0)	9.0 (0.0–112.2)	<0.001
Follow-up interval (years)	3.0 (2.0–3.8)	3.0 (2.1–3.9)	2.9 (2.0–4.0)	2.8 (1.8–3.4)	2.9 (2.1–3.6)	0.132

BMI, body mass index; WC, waist circumference; BP, blood pressure; FPG, fasting plasma glucose; HbA1c, haemoglobin A1c; TG, triglyceride; LDL-C, low-density lipoprotein cholesterol; HDL-C, high-density lipoprotein cholesterol; AST, aspartate aminotransferase; ALT, alanine aminotransferase; GGT, gamma-glutamyltransferase; HsCRP, high-sensitivity C-reactive protein; ASCVD, atherosclerotic CVD; CAC, coronary artery calcification.

### Association between the presence of NAFLD and/or MetS and the baseline CAC score

**Figure 1:**
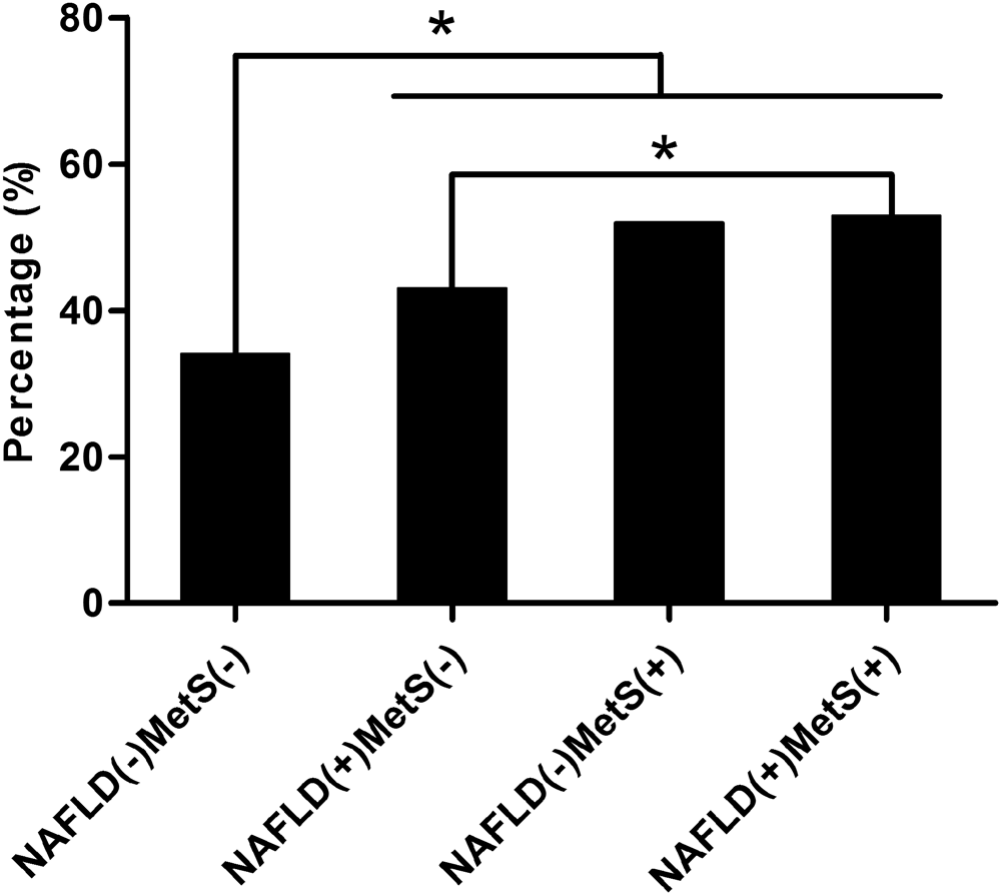
Proportion of the baseline coronary artery calcification score > 0 based on the presence of non-alcoholic fatty liver disease and metabolic syndrome. *P < 0.05.

The baseline CAC score in the NAFLD only group, the MetS only group and the group with both abnormalities was higher than that in the control group (Table 1). A baseline CAC score of > 0 was seen in 42.2% of the population. The proportion of subjects with a baseline CAC of > 0 was highest in the group with both abnormalities and lowest in the group without either abnormality (52.9% *vs*. 34.4%, respectively; Fig. 1). Both the NAFLD only and MetS only groups also had significantly higher proportions of detectable CAC at baseline than the control group (42.6% *vs*. 52.4% *vs*. 34.4%, respectively; Fig. 1).

Multiple logistic regression analysis was performed with the baseline CAC score as a dependent variable; odd ratios (ORs) and 95% confidence intervals (CIs) were calculated for the presence of NAFLD and/or MetS (Table 2). In an unadjusted model, subjects with NAFLD only (OR = 1.42, 95% CI = 1.05–1.91), MetS only (OR = 2.10, 95% CI = 1.37–3.22) and both NAFLD and MetS (OR = 2.14, 95% CI = 1.59–2.89) had significantly higher risks of detectable CAC than normal subjects (Table 2). However, the significance of these relationships in the NAFLD only and MetS only groups was lost after adjusting for age, sex, BMI, smoking, drinking, exercise habits, follow-up interval, LDL-C and hsCRP (Table 2).

**Table 2:** Association between the presence of NAFLD and/or MetS and baseline CAC score. OR for CAC score > 0 in reference with CAC score = 0.

	NAFLD(−)MetS(−)	NAFLD(+)MetS(−)	NAFLD(−)MetS(+)	NAFLD(+)MetS(+)
Crude OR	1.00 (Ref)	1.42 (1.05–1.91)	2.10 (1.37–3.22)	2.14 (1.59–2.89)
Model 1	1.00 (Ref)	1.13 (0.81–1.57)	1.65 (1.02–2.68)	1.45 (1.01–2.09)
Model 2	1.00 (Ref)	1.17 (0.83–1.63)	1.58 (0.97–2.57)	1.48 (1.02–2.15)
Model 3	1.00 (Ref)	1.14 (0.81–1.60)	1.54 (0.94–2.51)	1.45 (1.00–2.11)

Model 1 was adjusted for age, sex and BMI.

Model 2 was adjusted for the variables included in model 1 plus smoking, drinking and exercise habits.

Model 3 was adjusted for the variables included in model 2 plus follow-up interval, LDL-C and hsCRP.

### Baseline characteristics of the participants based on the CAC score progression

Compared with non-progressors, progressors were significantly older and demonstrated higher BMI, WC and systolic and diastolic blood pressure (BP). In addition, progressors were more likely to be male, current smokers, frequent drinkers and less physically active. In addition, progressors had a less favourable risk profile, which included higher prevalence of hypertension and diabetes and higher levels of FPG, HbA1c, TG, uric acid, AST, ALT, GGT and hsCRP. The 10-year FRS and 10-year ASCVD risk scores in progressors were also significantly higher than those in non-progressors. Progressors had higher baseline CAC scores and tended to be followed-up for a longer period (Table 3).

**Table 3:** Baseline clinical and biochemical characteristics based on the CAC score progression.

	Total	Non-progressor	Progressor	P
N (%)	1,173	881	292	
Age (years)	54.1 ± 7.4	53.4 ± 7.1	56.3 ± 8.0	<0.001
Sex (% male)	81.5	78.1	91.8	<0.001
BMI (kg/m^2^)	25.0 ± 3.0	24.8 ± 3.1	25.4 ± 2.5	0.002
WC (cm)	87.0 ± 8.2	86.2 ± 8.4	89.1 ± 7.3	<0.001
Systolic BP (mmHg)	119.5 ± 12.9	118.5 ± 12.4	122.4 ± 13.7	<0.001
Diastolic BP (mmHg)	76.6 ± 10.6	76.1 ± 10.4	78.3 ± 10.9	0.002
Current smoker (%)	27.4	25.2	33.9	0.004
Moderate drinker (%)	53.1	51.4	58.2	0.044
Physically active (%)	43.6	42.2	47.9	0.088
Family history of diabetes (%)	24.0	23.6	25.3	0.548
Diabetes (n, %)	155 (13.2)	98 (11.1)	57 (19.5)	<0.001
Hypertension (n, %)	393 (33.5)	262 (29.7)	131 (44.9)	<0.001
FPG (mmol/L)	5.8 ± 1.0	5.7 ± 1.0	6.0 ± 1.1	0.001
HbA1c (%)	5.5 (5.3–5.9)	5.5 (5.3–5.8)	5.6 (5.3–6.0)	0.003
Total cholesterol (mmol/L)	5.2 ± 0.8	5.2 ± 0.8	5.1 ± 0.9	0.590
TG (mmol/L)	1.3 (1.0–1.8)	1.3 (0.9–1.8)	1.4 (1.0–1.9)	0.027
LDL-C (mmol/L)	3.3 ± 0.7	3.3 ± 0.7	3.3 ± 0.7	0.747
HDL-C (mmol/L)	1.3 ± 0.3	1.4 ± 0.3	1.3 ± 0.3	0.003
Uric acid (µmol/L)	345.9 ± 83.0	341.5 ± 83.6	359.0 ± 80.0	0.002
AST (U/L)	25 (22–31)	25 (21–31)	27 (23–33)	0.002
ALT (U/L)	23 (17–31)	22 (17–31)	24 (19–34)	0.002
GGT (U/L)	25 (17–40)	24 (16–38)	30 (20–43)	<0.001
HsCRP (mg/L)	0.6 (0.3–1.3)	0.6 (0.3–1.3)	0.7 (0.4–1.4)	0.100
10-year FRS (%)	6.0 (3.0–10.0)	6.0 (2.0–10.0)	10.0 (6.0–12.0)	<0.001
10-year ASCVD risk score (%)	5.5 (2.7–9.7)	4.7 (2.3–8.7)	8.0 (4.8–13.4)	<0.001
Baseline CAC score	0 (0–21)	0 (0–10)	11 (0–93)	<0.001
Last follow-up CAC score	1 (0–48)	0 (0–15)	64 (10–226)	<0.001
Baseline CAC score category				<0.001
0 (n, %)		573 (65.0)	105 (36.0)	
>0 (n, %)		308 (35.0)	187 (64.1)	
Follow-up interval (years)	3.0 (2.0–3.8)	2.9 (2.0–3.6)	3.1 (2.4–4.0)	<0.001

BMI, body mass index; WC, waist circumference; BP, blood pressure; FPG, fasting plasma glucose; HbA1c, haemoglobin A1c; TG, triglyceride; LDL-C, low-density lipoprotein cholesterol; HDL-C, high-density lipoprotein cholesterol; AST, aspartate aminotransferase; ALT, alanine aminotransferase; GGT, gamma-glutamyltransferase; HsCRP, high-sensitivity C-reactive protein; ASCVD, atherosclerotic CVD; CAC, coronary artery calcification.

### Association between the presence of NAFLD and/or MetS and the CAC score progression

**Figure 2:**
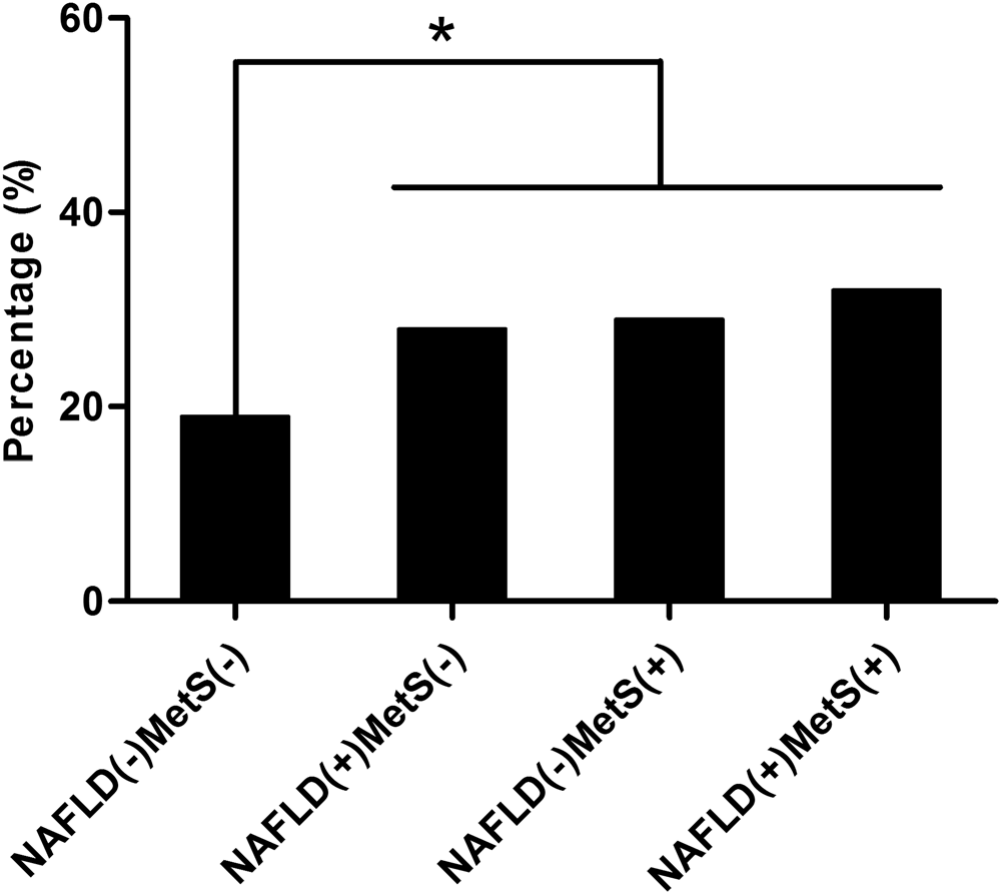
Proportion of the coronary artery calcification score progression based on the presence of non-alcoholic fatty liver disease and metabolic syndrome. *P < 0.05.

The proportion of subjects showing CAC score progression in the NAFLD only group, the MetS only group and the group with both abnormalities was significantly higher than that in the control group (28.3%, 29.1%, 32.0% and 18.6%, respectively) (Fig. 2). A statistically significant difference was found between the control and the other groups, but not among the three groups with abnormalities (Fig. 2).

Multiple logistic regression analysis was performed to compare the association of CAC progression with NAFLD and/or MetS and the results are shown in Table 4. In an unadjusted model including the whole population, the NAFLD only group (OR = 1.73, 95% CI = 1.22–2.43), the MetS group (OR = 1.80, 95% CI = 1.11–2.90) and the group with both abnormalities (OR = 2.05, 95% CI = 1.47–2.88) all showed significantly higher risks of progression of CAC than the control group (Table 4). Although the MetS only group did not show CAC progression (OR = 1.54, 95% CI = 0.90–2.63) in a model adjusted for age, sex, BMI, smoking, drinking, exercise habits, baseline CAC score, follow-up interval, LDL-C and hsCRP, the risk of CAC progression in the NAFLD only group and the group with both abnormalities was attenuated but remained statistically significant (OR = 1.53, 95% CI = 1.05–2.23 for the NAFLD only group; OR = 1.76, 95% CI = 1.18–2.62 for the group with both NAFLD and MetS) (Table 4).

**Table 4:** Association between the presence of NAFLD and/or MetS and CAC score progression.

	NAFLD(−)MetS(−)	NAFLD(+)MetS(−)	NAFLD(−)MetS(+)	NAFLD(+)MetS(+)
Crude OR	1.00 (Ref)	1.73 (1.22–2.43)	1.80 (1.11–2.90)	2.05 (1.47–2.88)
Model 1	1.00 (Ref)	1.55 (1.08–2.22)	1.56 (0.94–2.61)	1.74 (1.18–2.55)
Model 2	1.00 (Ref)	1.57 (1.09–2.26)	1.48 (0.88–2.48)	1.72 (1.17–2.55)
Model 3	1.00 (Ref)	1.53 (1.05–2.23)	1.54 (0.90–2.63)	1.76 (1.18–2.62)

Model 1 was adjusted for age, sex and BMI.

Model 2 was adjusted for the variables included in model 1 plus smoking, drinking and exercise habits.

Model 3 was adjusted for the variables included in model 2 plus baseline CAC score, follow-up interval, LDL-C and hsCRP.

## Discussion

In the present study, we examined the association between NAFLD, MetS and the progression of CAC in the asymptomatic, middle-aged, Korean population. Although the baseline CAC score was not significantly different, subjects with NAFLD but not MetS had a significantly higher risk of CAC progression compared with the subjects in the control group. We observed that a higher proportion of participants showed CAC progression in the NAFLD only group (28.3%) compared with the control group (18.6%) (Fig. 2). In a logistic regression analysis, the risk of CAC progression in subjects with both NAFLD and MetS was significantly higher than that in the normal subjects (OR = 1.76, 95% CI = 1.18–2.62). More importantly, the subjects with NAFLD but not MetS also had a significantly higher risk of CAC progression (OR = 1.53, 95% CI = 1.05–2.23), whereas the subjects with MetS only did not show significant CAC progression (OR = 1.54, 95% CI = 0.90–2.63) (Table 4). Our results indicated that NAFLD was closely associated with CAC progression, irrespective of the presence of MetS.

Previous studies reported a higher risk of CAC in subjects with NAFLD^5,7,9,10^ and, recently, a meta-analysis showed that patients with NAFLD had a higher risk of CAC than subjects without NAFLD based on multivariable-adjusted estimates^8^. However, the present study did not show a significant association between the presence of NAFLD only and the baseline CAC score after adjusting for other risk factors (Table 2), which appears to conflict with prior studies reporting an independent association after adjusting for confounding variables. A possible explanation is that our population mainly included low-risk participants because we recruited our participants during their routine health examinations and excluded those with a history of CVD. Our population included only 11.3% participants with a CAC score higher than 100 at the initial examination (data not shown). Therefore, this characteristic of our population might attenuate the power of the study to discriminate differences in baseline CAC score among the groups.

Although the baseline CAC score, measured by MDCT, has been established as a surrogate marker for coronary atherosclerosis, recent studies have shown that CAC score progression is significantly associated with higher risk of future cardiovascular events and all-cause mortality and represents a useful predictor of future cardiac events; it has, therefore, been proposed for use in the assessment of the effectiveness of medical therapies^5,11^. Considering that atherosclerosis progression is a dynamic and continuous process, monitoring of CAC progression using serial CAC scanning may be a more useful predictor of a patient’s risk of future events than the baseline CAC score^12^. In light of these findings, we assessed the CAC score progression using serial MDCT scans, which were performed a mean of 3 years apart. Interestingly, subjects with NAFLD only showed a significantly higher risk for CAC progression. These results indicate that NAFLD *per se* still has a significant relationship with CAC progression even after adjusting for known metabolic factors as confounders.

Although the pathophysiological mechanisms on how NAFLD affects CAC progression cannot be elucidated based on the results of this study, several plausible mechanisms have been suggested^13–19^. Endothelial dysfunction of the systemic circulation, the first step in the process of coronary atherosclerosis, has been observed in NAFLD^13,14^. In addition, several studies have demonstrated a positive association between liver fat and prothrombotic factors, including factors VII, IX, XI and XII and the plasminogen activator inhibitor-1^15,16^. This procoagulant imbalance in NAFLD may thus represent a causative link between NAFLD and CVD. Greater oxidative stress might also explain the high cardiovascular risk associated with NAFLD. Plasma homocysteine is a cardiovascular risk factor because of its adverse effects on cardiovascular endothelium and smooth muscle cells and high levels of plasma homocysteine have been consistently reported in NAFLD^17,18^. Finally, because the liver contains the largest number of macrophages and immune cells, cytokines secreted by the injured liver have been proposed to be one of the major pathogenic mechanisms generating systemic inflammation that leads to CVD^19^. Although pathophysiological mechanisms were not investigated in this study, the mechanisms described above could provide explanations for the association between NAFLD and CAC progression.

The most novel finding of this study is the independent association between NAFLD and CAC progression in individuals without MetS. Recently, a large meta-analysis of observational studies indicated that NAFLD is significantly associated with a higher risk of fatal and non-fatal CVD events^20^. However, whether NAFLD is associated with higher risk for CVD beyond the conventional cardiovascular risk factors and co-morbidities such as MetS remains uncertain. Previous studies have shown that individuals with MetS have a higher risk of cardiovascular morbidity and mortality^21^. Jelavic *et al*. demonstrated that MetS by National Cholesterol Education Program (NCEP) Adult Treatment Panel III (ATP III) as a pathophysiological concept is relevant and superior to its components in risk prediction of patients with acute ST elevation myocardial infarction urgently treated with primary percutaneous coronary intervention^22^. Furthermore, MetS was found to be an independent predictor of the rapid development or progression of CAC in a large retrospective longitudinal study^23^. NAFLD and MetS share common pathophysiological pathways and risk factors, including central obesity, hypertension, dyslipidemia and dysglycaemia^24^. The homeostatic model assessment of insulin resistance and hsCRP were reported to be independently associated with fatty liver index, implying that insulin resistance and subclinical inflammation have important roles in NAFLD and MetS^25,26^. In addition to these conventional CVD risk factors, hypercoagulation, impaired fibrinolysis, obstructive sleep apnoea, hyperuricaemia and polycystic ovary syndrome are frequently present in both NAFLD and MetS^24^. Furthermore, common therapeutic approaches, including lifestyle intervention, some anti-obesity and anti-diabetic medications and statins are beneficial for both NAFLD and MetS^24^. Thus, the common pathophysiology, risk factors and therapeutic approaches support that NAFLD is regarded as a hepatic manifestation of MetS^24^. Taking these findings together, it remains unclear whether NAFLD affects cardiac outcomes through the effects of metabolic risk factors it shares with MetS or NAFLD alone. Thus, clarification of whether NAFLD *per se* has an independent association with CVD is important. The present study is the first to show that subjects with NAFLD only, without MetS, have a higher risk of CAC progression than healthy subjects. This result suggested that NAFLD could have a harmful effect on the cardiovascular system, irrespective of the presence of MetS. Therefore, detecting and treating NAFLD in metabolically healthy patients are important.

Our findings implied that the presence of NAFLD or MetS in healthy population represented a risk factor for atherosclerosis and future CVD, implying that CV risk factors should be treated in these individuals. In patients with MetS, beyond lifestyle therapies directed toward underlying risk factors, attention must be given to metabolic risk factors, including hypertension and atherogenic dyslipidemia^27^. The joint guidelines of the European Association for the Study of the Liver, European Association for the Study of Diabetes and European Association for the Study of Obesity and a recent expert panel statement both suggest lifestyle management and statins for NAFLD to decrease LDL-C and CVD risk^28,29^. Dyslipidaemia is frequently associated with NAFLD, and patients with NASH have increased levels of small, dense LDL3 and LDL4 compared with those with simple steatosis; LDL3 and LDL4 implicate the crucial role of dyslipidaemia for CVD in NAFLD^30,31^. Although there is concern that patients with NAFLD and dyslipidaemia could develop liver enzyme elevation, evidence from previous studies showed that statins can be used safely to treat dyslipidaemia in patients with NAFLD^30^. Furthermore, in some studies, reduction and/or normalisation of liver enzymes due to statin use has been observed^30,32–35^.

Several therapeutic interventions for NAFLD, including anti-diabetic medications, have been proposed^36^. Rizvi *et al*. reported that liraglutide significantly reduced carotid IMT, a surrogate marker of atherosclerosis, independently of glucometabolic changes in diabetic subjects with NAFLD^37^. A recent meta-analysis has found a significant improvement of NAFLD in patients with type 2 diabetes mellitus treated with incretin-based therapies^38^. However, Smits *et al*. reported the conflicting data that hepatic fat contents and hepatic fibrosis scores were not altered by liraglutide or sitagliptin^39^. However, there are conflicting results regarding the effects of incretin-based therapies on NAFLD^39,40^. These discrepancies could be partly caused by the difference of statin use in study subjects, which can influence the interpretation of the results because statins can improve NAFLD^40^. Apart from anti-diabetic drugs, NAFLD/NASH may be improved in terms of both biochemical and histological features by statins^40–42^. Taken all previous findings together, statin treatment may be beneficial in patients with NAFLD.

This study had several limitations. First, this was a retrospective analysis without a histopathological investigation; therefore, causality cannot be established. However, the previously reported pathological mechanisms that underlie the relationship between NAFLD and CVD might explain our findings. Second, we could not obtain quantitative histories of alcohol consumption of the participants; therefore, we could not discriminate between NAFLD and alcoholic FLD. Third, prescribing statins after study enrolment was not considered in the analyses and these drugs might have contributed to the calcification of coronary plaques^43^. Finally, the definition of CAC progression we used might be problematic because there is no consensus regarding this yet^44^. Most previous studies assessed CAC progression by measuring absolute changes in CAC the scores between baseline and follow-up^6,44^ or mean changes in the square-root-transformed (SQRT) method^5,45^. The large number of zeros and skewed distribution of changes in CAC may also have compromised the precision of the cut-off value used to define progression^44^. However, the best CAC progression model for the prediction of mortality has been shown to be the SQRT method, which we chose to use in this study and a SQRT difference of 2.5 provides the best fit for the data^5^.

In conclusion, this study was the first to demonstrate that NAFLD is an independent contributor to CAC progression, irrespective of the presence of MetS. Our data suggest that special attention should be paid to those individuals with NAFLD but not MetS because they are at high risk for the development of CVD.

## Methods

### Ethics statement

In accordance with the ethical guidelines of the declaration of Helsinki and Korea Good Clinical Practice, this study was approved by the institutional review board of the Asan Medical Center (AMC). All participants provided written informed consent.

### Study population

The study population consisted of 7,300 participants who underwent baseline coronary computed tomography angiography (CCTA) using a 64-slice MDCT scanner during routine health evaluation at AMC (Seoul, Republic of Korea) between January 2007 and June 2011. Of these, repeat CCTA was performed on 1,591 participants until December 2014. This analysis also used data obtained using in-person follow-up examinations conducted after the baseline examinations. Each participant completed a questionnaire that listed a history of previous medical and/or surgical diseases, medications and drinking and smoking habits. The drinking habits were categorised based on frequency (1 or 2 times/week [moderate drinker]), the smoking habits as non-current or current and the exercise habits based on frequency (2 or 3 times/week [physically active])^46^. A history of CVD was recorded based on each participant’s history of physician-diagnosed angina, myocardial infarction and/or cerebrovascular accidents. Participants with an FPG of ≥7.0 mmol/L and/or HbA1c level of ≥6.5% were categorised as diabetic^47^. In addition, participants who reported the use of anti-diabetic medications on a self-report questionnaire were considered to have diabetes^48^. Hypertension was recorded if subjects had a systolic and/or diastolic BP of ≥140/90 mmHg or if they were receiving anti-hypertensive medications. The 10-year FRS and 10-year ASCVD risk score were calculated to estimate the cardiovascular risk^49^. The 10-year ASCVD risk was estimated using the Pooled Cohort Equations for non-Hispanic whites, which was developed by the Risk Assessment Work Group^50^.

Participants with a history of CVD at baseline examination (n = 95) and those that were receiving statins (n = 238) were excluded. Participants who underwent percutaneous coronary intervention (n = 8) or coronary arterial bypass surgery (n = 3) after the initial examination were also excluded. Subjects that were not between 20 and 79 years were also excluded (n = 3). Finally, participants with hepatitis B surface antigen (n = 48), positive hepatitis C antibody test (n = 19) and liver cirrhosis or hepatocellular carcinoma (n = 4), as well as recipients of liver transplantation (n = 2), were excluded. Some participants met more than two exclusion criteria. After excluding ineligible subjects, 1,173 subjects, with a mean age of 54.1 years (range, 33–79 years), were enrolled in the final study population.

### Clinical and laboratory measurements

Height and body mass were measured with the participants wearing light clothing and no shoes. BMI was calculated as body mass in kilograms divided by the square of the height in meters. WC (in cm) was measured mid-way between the costal margin and the iliac crest at the end of normal expiration. BP was measured on the right arm after resting for 5 min using an automatic manometer with an appropriate cuff size. After overnight fasting, early-morning blood samples were drawn from the antecubital vein into vacuum tubes and subsequently analysed by the central, certified laboratory at AMC. Measurements included concentrations of fasting glucose, insulin, hsCRP, several lipid parameters and liver enzymes.

Fasting total cholesterol, HDL-C, LDL-C, TG, uric acid, AST and ALT levels were measured using enzymatic colorimetric methods on a Toshiba 200FR Neo analyser (Toshiba Medical System Co., Ltd.). GGT was measured using the L-g-glutamyl-p-nitroanilide method (Toshiba Medical System Co., Ltd.). FPG and hsCRP were measured using the enzymatic colorimetric method on the Toshiba 200 FR auto-analyser and the immunoturbidimetric method (Toshiba Medical System Co., Ltd.), respectively. Ion-exchange high-performance liquid chromatography (Bio-Rad Laboratories, Inc., Hercules, CA, USA) was used to measure the HbA1c levels. All enzyme activities were measured at 37 °C.

### Definitions of NAFLD and MetS

Hepatic ultrasonography was performed to diagnose NAFLD (Ultrasound Systems IU22; Philips, Holland) by experienced radiologists who were blinded to the laboratory and clinical details of the study subjects at the time of the procedure. Fatty liver was diagnosed based on the characteristic ultrasonographic features that were consistent with ‘bright liver’ and evident contrast between hepatic and renal parenchyma, vessel blurring, focal sparing and narrowing of the lumen of the hepatic veins^1^.

MetS was defined based on the criteria established by the NCEP-ATP III using Asian-specific cut-off points for abdominal obesity, as recommended in the criteria^51,52^. An individual was classified as having MetS if the following five criteria were met: (1) WC of ≥90 cm in men and ≥80 cm in women; (2) TG ≥150 mg/dL (1.7 mmol/L); (3) HDL-C of < 40 mg/dL (1.0 mmol/L) in men and < 50 mg/dL (1.3 mmol/L) in women; (4) BP ≥130/85 mmHg or the use of anti-hypertensive medication and (5) fasting glucose ≥100 mg/dL (6.1 mmol/L) or the self-reported use of anti-diabetic medication (insulin or oral agents).

### Use of MDCT to assess the CAC score

MDCT examinations were performed using either 64-slice, single-source, CT (LightSpeed VCT; GE, Milwaukee, WI, USA) or dual-source CT (Somatom Definition or Somatom Definition Flash; Siemens, Erlangen, Germany)^53,54^. The CAC score was calculated using an automated software program and the Agatston scoring method^55^ and the participants were categorised based on the cut-off points used by Greenland *et al*.^56^ (none, 0; mild, 1–100; moderate, 101–300; severe, > 300).

CAC progression was defined as (1) incident CAC, indicating a baseline Agatston score of zero but detectable CAC at the follow-up examination in a population free of CAC at baseline^57,58^ or (2) an increase of ≥2.5 units between the baseline and the final square root of CAC scores in participants with detectable CAC at baseline^5,59,60^. To eliminate the dependence of residual inter-scan variability on the baseline CAC score, square root transformation of the CAC score was performed before the estimation of CAC progression. Using the data published by Hokanson *et al*., ‘progressors’ were defined as individuals with a difference of ≥2.5 units between the baseline and the final square root of their CAC scores (the SQRT method)^5,59,60^. Expressed differently, a change of < 2.5 units between the baseline and the final square root of the CAC score was considered to be within the margin of error for CAC score estimation using MDCT, and this was thus attributed to inter-scan variability. Such participants were classified as ‘non-progressors’^5,59,60^.

### Statistical analysis

Continuous variables with normal distribution were expressed as mean ± standard deviation, whereas continuous variables with skewed distribution were expressed as median (and interquartile range). Categorical variables were expressed as percentage. In the comparison of sub-groups reflecting the presence of NAFLD and/or MetS, one-way analysis of variance with Scheffé’s method and the Kruskal–Wallis test with the Dunn procedure were used to assess continuous variables and the Chi-squared test was used to assess categorical variables. The demographic and biochemical characteristics of the sub-groups categorised by CAC score progression were compared using the Student’s *t*-test or the Mann–Whitney *U* test for continuous variables or the Chi-squared test for categorical variables. Logistic regression analysis was performed to calculate the ORs and 95% CIs of the sub-groups defined by the presence of NAFLD and/or MetS to predict the baseline CAC and CAC progression. All statistical analyses were performed using SPSS software (version 20.0 for Windows; SPSS, Inc., Chicago, IL, USA). In the present analyses, a two-sided *P*-value was adopted and *P* < 0.05 was considered to be statistically significant.

## Data Availability

The datasets generated during and/or analysed during the current study are available from the corresponding author on reasonable request.
